# Advance care planning in Norwegian nursing homes – limited awareness of the residents’ preferences and values? A qualitative study

**DOI:** 10.1186/s12877-019-1378-6

**Published:** 2019-12-23

**Authors:** Lisbeth Thoresen, Reidar Pedersen, Lillian Lillemoen, Elisabeth Gjerberg, Reidun Førde

**Affiliations:** 1 Department of Interdisciplinary Health Sciences, Harald Schjelderups hus Forskningsveien 3a/2b, Post Box 1089, 0373 Oslo, Norway; 2 Centre for Medical Ethics, University of Oslo, Oslo, Norway

**Keywords:** Advance care planning, Nursing home, End of life, Communication, Qualitative

## Abstract

**Background:**

52% of all deaths in Norway occur in nursing homes. Still advance care planning (ACP) is scarce and heterogeneous. To improve the implementation and practice of ACP in nursing homes, knowledge about health care professionals’ views on ACP is vital. The objective of this study is to explore nurses and physicians’ aims and experiences with carrying out ACP in nursing homes.

**Methods:**

Semi-structured group interviews were conducted with 20 health care professionals, recruited from nursing homes where ACP was performed regularly. Qualitative content analysis was used to analyse the data.

**Results:**

The primary aim of the nursing home professionals when doing ACP in nursing homes were to build alliances with next of kin to avoid misunderstandings and future conflicts. Two main experiences with ACP were described: i) due to the sensitivity of ACP issues, it was important to balance directness with being sensitive, and ii) when the physicians raised questions concerning future medical treatment, the answers from residents as well as next of kin were often hesitant and unclear.

**Conclusion:**

Our study add insights into how ACP is practiced in nursing homes and the professionals’ agenda. A focus on medical issues and achieving consensus with next of kin may result in lack of involvement of the residents and limited awareness of the residents’ needs. Interdisciplinary approaches, ACP-training and tailored guidelines may improve the implementation and practice of ACP.

## Background

Enabling patients to express their goals and preferences regarding future medical treatment and care is emphasized in health care, not the least in geriatrics and palliative care [1–3]. In Norway, white papers as well as legal rights state the patients’ right to participate in health care decisions [4]. These rights are particularly strong when death is approaching. Thus, an official guideline on decision-making processes in the limitation of life-prolonging treatment recommends ACP, also in nursing homes (NH) [5]. Although 52% of all deaths in Norway occur in NHs, (numbers from 2017, [6]), ACP is scarce and heterogeneous here [7]. This may be related to the high numbers of NH residents with some degree of cognitive impairment (close to 80%) [8], and there are discussions about whether ACP conversations should be initiated in dementia care [3, 9]. Professional care for people with dementia has changed over the last decades, from mainly meeting basic needs, to acknowledging the individual person and his or her particular worth and preferences [10]. Today, person-centered care [11], also involving decision-making for people with dementia [10] is acknowledged and recommended [12, 13]. However, the implementation and practice of ACP in NHs is described as a “worldwide challenge” [14]. Furthermore, we have limited knowledge of NH professionals’ aims and experiences with carrying out ACP in “ordinary” NHs- i.e. NHs where there has been little or no systematic ACP implementation or training. Such knowledge is probably vital to improve the implementation and practice of ACP.

### Aim

The objective of this study is to explore nurses and physicians’ aims and experiences of carrying out ACP in ordinary nursing homes. The present study is a first step in a larger research project, aiming to improve the autonomy and dignity of NHs residents suffering from life-threatening illnesses and dementia through better implementation of ACP [15].

## Methods

To gather rich data, qualitative group interviews with experienced health professionals were carried out in NHs that had started up ACP on a regular basis.

### Setting

The 850 nursing homes in Norway are part of the public health care system, and run by the municipalities. The population of Norway is 5.3 million people, and in 2018, 32,000 elderly persons permanently lived in NHs. Of persons aged 80 years and older, around 13% live in a NH, but this group makes up around 80% of the NHs population [16]. Mean age for persons who died in NHs in 2017 was 87,5 years old [17]. Women make up around 70% of the NHs population [18]. The low grade of institutionalization in Norway mirrors that independent living at home, as long as possible, is the ideological ideal, and influences health policy strategies. As a result, people are old and in need of extensive care and support when they move from home to a NH. Frailty, vulnerability, disability and multiple diagnoses [19, 20] characterize the NH population. Staff members are nurses, nurse assistants and physicians. There are large variations between municipalities and NHs when it comes to physician employment, ranging from full-time positions to GPs working 20% as medical supervisors [21].

### Recruiting NHs and developing data

While recruiting nursing homes for the study in 2014, we tried to identify NHs where health care professionals had some experience with ACP; where ACP had been practiced for at least 1 year. This turned out to be difficult, but in the end, we recruited eight NH wards (labelled as wards A, B, C…H). In these wards, we observed ACP conversations between NH’ staff, residents and/or next of kin [22]. Only in one of the conversations was the resident not present, due to severe dementia. The participating residents where assessed by the health care professionals to be competent. However, two of the residents had aphasia which no doubt made the conversations challenging. Next of kin were present in all conversations. The content of the observed ACP conversation was primarily related to questions concerning future medical treatment, hospital admission and DNR. After the conversations, we interviewed the resident and/or next of kin about their experiences of participating in ACP [23]. The interview guide was developed by all authors [24] (Additional file 1). The present study is based on seven group interviews with 20 NH staff (11 nurses, 8 physicians, 1 nursing assistant, and 1 nursing student) who had participated in the observed ACP conversations. For practical reasons, two to five staff members were interviewed together. Besides centering on what had taken place during the conversations, a semi-structured interview guide guided the interviews, covering issues like what staff members perceived as the aim of the conversation, and how they experienced the residents’ participation. The interviews were audio recorded and transcribed verbatim by the first author.

### Data analysis

To analyse the entire data set, content analysis, as described by Schreier [25] was carried out. This approach, consisting of generating, defining and evaluating categories, is useful for identifying aspects that relate to the research questions, and for providing a good description of the material [25]. As a start, all authors read the transcribed interviews and discussed analytical ideas, thereby identifying the main categories. By main categories, we understand aspects of the material, which relate to or answer the study questions. The main categories were later systematically refined by the first author by generating subcategories which “specify what is said in the material with respect to these main categories” [25]. Creating subcategories means coding, that is, segments of individual staff members’ responses and answers during interviews have been ‘named’ and sorted. Generation of subcategories and naming of main categories have been discussed by all authors. Quotes illustrate themes in the results section, and are referred to by letter (NHs wards) and number.

### Ethical considerations

Permission was granted from the head of the NH and from the Data Protection Official for the University of Oslo (NSD 37368). The study did not require approval from Regional committee for medical and health research ethics (2013/19937). Staff members were informed in writing and orally about the study, and asked if they wanted to participate. All agreed and gave their written consent.

## Results

The analysis of the interviews generated the following themes: The primary aim of advance care planning is to build alliances with next of kin to avoid misunderstandings and future conflicts. Two main experiences with ACP were described: i) due to the sensitivity of ACP issues, it was important to balance directness with sensitivity during the conversations. ii) the answers from residents as well as next of kin regarding future treatment were often hesitant and unclear.

### The primary aim of ACP: to build alliances with next of kin to avoid misunderstandings and future conflicts

Even though residents and next of kin were asked about future preferences, exploring resident preferences was not the main motivation for facilitating conversations concerning end-of-life care. Instead, nearly all participants described that the most important aim was to *build alliances* and come to some kind of agreement on future treatment with next of kin. The use of metaphors such as *team, supporters* and *walking together* (B44, E25, G18) underlines how much emphasis staff put on agreement with next of kin in order to *avoid conflicts* (G18), illuminated by the following quote: *This is a conversation where we present how we see the resident’s state, and where we present how we think about medical treatment. I present some arguments about why we should change from active to palliative care…The aim is to find, or come to a common view about the resident, with the next of kin. To us, everything is logical and clear, but family members do not necessarily think the way we do. The conversation prevents misunderstandings and accusations between staff and next of kin when the time comes. We ask whether they agree with our understanding* (C5, C9, C40).

Obtaining shared views could, however, take some time. Next of kin were described as needing *time to digest information* (F25), and as *‘ready’* if *they have seen the same as us, have the same thoughts* (C23). However, some family members never accept that their loved ones are dying: *you may have told them, but the message is not received…it is difficult if your view is not shared with close family members. Some need a long time even if the time is limited* (D 44).

The staff also found disagreement among next of kin difficult to handle: *you are talking to one family member today, and another tomorrow, and he or she might think quite differently. That is a challenge. It is essential to talk to the right person* (E26). According to one physician, next of kin could also have problems understanding their role and responsibility in the decision-making process: …*and we talk to them (next of kin) face to face, and explain what their role in the decision-making hierarchy is and who makes the final decision. We find that some become somewhat unsure about their role; they become angry and we often have many discussions with them. This might lead to a difficult terminal phase; it can become a nightmare* (B40).

### Main experiences with ACP

#### Balancing directness with sensitivity

The physicians described how they had developed their own, personal way of raising the question about future life-prolonging treatment, shaped by experiences and feedback from next of kin: *My experience is that next of kin appreciate that physicians are direct and concrete because many health care professionals are not. They like that someone dares to talk about it* (E39). And: *I want it to be clear what we are talking about; no one should wonder after the ACP-conversation; what were we talking about? I find that people appreciate that I am clear* (A36). However, being direct and clear had to be balanced with sensitivity and openness to diversity: *Residents are very diverse people, and some know what they want and some do not* (G9). Physicians described how they tried to *perceive the situation: sense where the resident and next of kin ‘are’,* and *feel how the conversation is going* (G2, A35). The questions that residents had to answer were described as *sensitive* (B62), and one physician talked about this *heavy stuff* when reflecting on what kind of information he needed from residents (B56).

In general, all staff members expressed that they found being part of and facilitating advance care planning *stressful, hard and demanding*, but particularly for the physicians (B16, F78, D 36). Even if the physicians had years of experience with communication about end-of-life care, they expressed how ACP *takes a lot of doing* (G33) and how one *feels put out of action in such situations* (F27).

#### Hesitant and unclear answers

The staff members shared the view that clear preferences or wishes from the residents who participated in ACP were rare. Particularly when residents were asked if they wanted to go to the hospital in the case of serious illness, or when they were asked questions about future medical treatment, answers from residents could be hesitant and unclear. The professionals told that: *Often, we do not get clear answers from these conversations* (F6). *Some have clear opinions about end-of-life care, but in this conversation; she did not tell us much about what she wanted. She wanted to be safe and to be cared for, but she did not have any opinions yet* (G9, G14). Interestingly, even if the resident in the last quote expresses views concerning her well-being, it seems like this is not understood as relevant for staff, who want ‘opinions’ on treatment alternatives.

According to the nursing home professionals, usually residents with mild dementia were involved in ACP, but one physician stated: *I am sorry to say, but patients are too ill and too impaired to take part in ACP. They are not able to. Our ambition is that they should take part in planning for the future, but it hasn’t happened once* (C32). In addition, residents with aphasia could be particularly challenging to understand: *First, I thought that she was positive, but then she started to cry which made me think – what does she want? I agree that her answer is in a grey area…it was difficult to read her* (A18–19). Trying to understand what the residents wanted, the staff would interpret residents’ *body language* (F10), facial expressions, as well as *moods* (B 23, D32). Hesitant and unclear answers also came from next of kin. The participants described next of kin as being in a very difficult position when asked for views and preferences on behalf of their loved ones: *it is not easy for them. They wonder if they have contributed to the right decision. They want to wait – they want to postpone the conversation about future decision-making until the dying process has started* (F31, F 80). And: *There are many people who haven’t discussed these matters, and as her husband said: we have never talked about this, and the son says that he has to think through what he would have wanted for himself. It is difficult to answer for a mother or a spouse. I think we have to accept that clear answers are rare* (C28).

## Discussion

Since relatively few Norwegian NHs practice ACP on a regular basis, it is of particular interest to understand the motives and experiences of health personnel who actually do this. The findings from this study provide in-depth knowledge that may help us to identify factors that may improve ACP in NHs.

### Aims and content of ACP

Our study indicates that the most prominent motive for raising the issue of end-of-life care with residents and their next of kin was to prevent future misunderstanding, disagreement and conflicts between staff and family members. If it was possible to establish a common view about the resident’s situation and best interests concerning life-prolonging treatment, misunderstandings and conflicts could be prevented. There may be good reasons for this way of thinking. Conflicts with next of kin, particularly during the last phase of the resident’s life, are uncomfortable [26] and ethically challenging [27, 28]. That health care professionals, and in particular the physicians, found it demanding and hard to discuss end-of-life care, may also explain why they focused mainly on medical issues. From the perspectives of family members, mutual understanding between next of kin and health care professionals improves their experiences when someone close to them is dying [29]. However, although ACP aims to improve residents’ involvement in health care, in this study, less attention is actually paid to what matters to the NH residents; to their interests and wishes, and their abilities to participate in ACP. As we see it, there are good reasons for considering new ways of involving frail, older persons living in NHs.

Giving clear answers of future treatment at the end of life may be challenging. ACP is one way to avoid not talking about these issues. However, a too strong focus on directness and medical information may contribute to more hesitant and unclear answers, and less, rather than more patient centeredness [22].

### Multidisciplinary ACP in NHs

Tensions concerning the aim and content of ACP may be related to how these conversations are organized and facilitated. From observing ACP conversations, we know that the physicians were the most active party, while the participating nurses were more passive [22]. The nurses did not take an active part in the exchange about future illness or dying in any way. In fact, one nurse claimed that she was happy to leave the difficult questions concerning end-of-life decision-making to the physicians. This has been described and discussed elsewhere [22]. This pattern was also present in the interviews, with physicians being quite active in describing and reflecting on how they carried out ACP, while the nurses contributed less. However, co-operation between health care professionals is considered important to improve resident involvement and increase the quality of ACP [30, 31]. Also, if we want ACP to cover broader aspects of the resident’s life; if we want to know more about the resident’s *overarching philosophies and priorities in life* [32] and the resident to become more involved in decision-making, other staff members should be involved in ACP. NH residents have daily, lengthy and often close contact with nurse assistants, unskilled staff, as well as the cleaning personnel. In this contact, ACP-relevant aspects may have been talked about and important relations may have developed. The possibility of involving these staff members should be explored. They may know what matters to the residents and can bring these insights into the ACP-process, contributing to a richer picture, also regarding planning for the future [33]. They may also understand more of what residents’ hesitance when it comes to expressing their views, actually is about, e.g. whether it is caused by the sensitive character of the issues or by cognitive impairment. This is particularly important when we know that many of the residents have dementia and reduced capacity, and that trust in staff members is important for successful ACP [33, 34]. Trusted staff and next of kin that knows the resident well may contribute to increase the resident decision making capacity and supported decision-making, rather than substituted decision-making. To improve the implementation and use of ACP in NHs, Gilissen et al. [14] suggests that all nursing home staff as well as volunteers receive training to help them recognize “triggers for ACP”, and how to engage in spontaneous ACP-related conversations. The importance of involving the whole ward in ACP is also described by Sævareid et al. [35].

### Training to improve ACP in NHs

End-of-life issues are sensitive [36], and there is a lack of educational programs teaching how to carry out such conversations. None of the participants in our study had been trained in end-of-life communication, advance care planning or how to involve residents or next of kin in ACP. The legal requirement and moral obligation to secure patient participation in health care require communication training programs, as well as the development of tools and meeting places, in order to build cultures where communication and decision-making processes are implemented and adapted locally [37]. Communication training may improve professionals’ self-efficacy and knowledge [38, 39], and should include reflections on the staff members’ own emotions [40]. Knowledge and availability of helpful pathways can contribute to reducing the burden on the staff [33, 40]. Training and guidelines may make it less stressful for residents and next of kin to participate, for example through emphasizing ACP as a process over time and that may include also more spontaneous conversation [15].

## Conclusion

Our study adds insight into how advance care planning is practiced in NHs, where the staff members have with little or no training and implementation support. We find that participants’ focus on medical issues and achieving consensus on the resident’s prognosis and treatment aims, may be accompanied by limited awareness of the individual resident’s needs, worries and hopes at the end of life. With blurry answers from residents and next of kin, this way of doing ACP hardly adds to the goal of increasing resident autonomy in end-of-life decisions. Interdisciplinary ACP may reduce the focus on medical issues and facilitate resident participation and supported decision-making. ACP is perceived as complex communication, and including residents with cognitive impairment makes these conversations even more challenging. Training and tailored guidelines may be useful to improve implementation and practice of ACP in NHs and to develop a broader understanding of the aims, content and possible outcome of ACP.

## Supplementary information

**Additional file 1.** Interview guide Thoresen.

## Data Availability

The dataset generated and analyzed during the current study is in Norwegian only. The anonymized dataset is available from the corresponding author on reasonable request.

## Abbreviations

ACP: Advance Care Planning
NH: Nursing Homes
