# Supplementary material for: Advance care planning in Norwegian nursing homes – limited awareness of the residents’ preferences and values? A qualitative study
Source: BMC Geriatr. 2019 Dec 23;19:363. doi: 10.1186/s12877-019-1378-6 (PMC6929496; doi:10.1186/s12877-019-1378-6)
Supplement: Supplementary file 1 — Additional file 1. Interview guide Thoresen. [file 12877_2019_1378_MOESM1_ESM.docx]

**Interview guide nursing home staff Thoresen 2014**

The interview with staff will take place shortly after an ACP conversation with one of the NH residents. The staff will be interviewed together, and all involved are invited to share their views and experiences on this or other ACP processes.

What are the aims of ACP conversations in this nursing home in general, and to this conversation in particular?

Tell me about the conversation we just finished:

- Who invited the resident and how? In written or oral? Are residents given the opportunity to reject the invitation? Do residents in general want to be part of ACP?
- How was relatives invited?
- Do you use a template in conversations? If yes, who is responsible for the template?
- Why did you, as staff members, participate? What is your role in the conversation?
- How did you start the conversation?
- How do you think about this conversation? Was it successful – or not? Was it something special with this one? Or was it similar to other ACP conversations? In which ways? How will you characterize the conversation?
- What did you talk about? What was most important? What was new to you? Any surprises? Was the aim of the conversation fulfilled?
- How do you handle information from the conversation? How is it documented? How will it affect future decision-making?
- What about the resident – how do you think she/he experienced taking part in ACP?
- And the relatives – their experiences?
- How do you understand patient involvement?
- Do residents change their wishes/opinions? How do you handle this?
- To who is this conversation important?
- How direct and open shall end-of-life issues be addressed?
- Tell me about an ACP conversation that was useful in later decision-making
- What are the main barriers to ACP in nursing homes?
